# Back to the Future: Can Vaccines Win the Long-Term Fight Against COVID-19?

**DOI:** 10.3389/fpubh.2022.929445

**Published:** 2022-06-13

**Authors:** Hildegund C. J. Ertl, Sue L. Currie, David M. Livermore

**Affiliations:** ^1^The Wistar Institute, Philadelphia, PA, United States; ^2^Virion Therapeutics, Newark, DE, United States; ^3^Norwich Medical School, University of East Anglia, Norwich, United Kingdom

**Keywords:** COVID-19, vaccinations, adenovirus vector vaccine, mRNA vaccine, public health strategies

## Introduction

The SARS-CoV-2 pandemic started in March 2020, and, to date, has killed over 6 million people. Vaccines were developed with remarkable speed and reduced the death toll. Notwithstanding, the pandemic endures, and accumulating viral mutations are progressively reducing vaccine efficacy, which is short-lived, even against the original virus. Thus, the UK is recording 4.9 million infections per week, despite evidence that 99% of its 68 million population have anti-SARS-CoV-2 antibodies. Before we embark on endless booster immunizations, it is time to review our strategies against COVID-19.

And, for that, we must go back to the beginning.

Globally, most countries are using mRNA or adenovirus (Ad) vector vaccines that to produce viral spike (S) protein, and to mount an immune response against it. In large (but brief) clinical trials, two-dose mRNA vaccines (Pfizer-BioNTech and Moderna) prevented symptomatic infections in >90% of recipients. The four Ad vector vaccines (i.e., the single-dose human serotype (HAdV-26) vector from Johnson & Johnson, the two-dose chimpanzee Ad vector from AstraZeneca; the two-dose Sputnik V vaccine, which primes with HAdV-26 and boosts with HAdV-5 (1) and the single-dose AdHu5 vaccine from Sino Biologics Inc) provided 63, 67, 92 and 63% protection, respectively, in phase III trials, also of short duration.

None of these vaccines induces sterilizing immunity and, whilst they reduce severe disease, hospitalization, and death, protection against infection and transmission is limited. For the mRNA vaccines, used in >95% of US vaccine recipients, protection against infection wanes over circa 6 months, prompting booster immunizations (2), which protect for only about 4 months (3). This precipitous loss in efficacy reflects declining antibody titers and the proliferation of more transmissible variants, notably omicron, which escape vaccine-induced antibodies. Attempts to induce variant-specific immunity by updating the mRNA vaccines' insert have met limited success (4) due to the growth advantage of vaccine- or infection-induced memory B cells against an early SARS-CoV-2 isolate over naïve B cells specific for new epitopes of the mutated virus–a phenomenon dubbed “original antigenic sin.”

The wide preference for mRNA vaccines reflected their initial high efficacy and safety profile; Ad vector vaccines appeared less protective and were linked to thrombotic thrombocytopenia, a rare and potentially fatal adverse event. Recent evidence, however, suggests that Ad vectors may induce more durable antibody responses. Coupled with lower acquisition cost and better heat stability, this may confer advantage over mRNA vaccines (5).

Uncertainty on the relative merits of mRNA and Ad vaccines for providing prolonged protection against severe disease in the elderly/vulnerable populations–who most direly need to be vaccinated–reflects the early emphasis on developing and distributing vaccines as quickly as possible. Consequently, there was limited effort on optimizing regimens, assessing immunological correlates of protection, or identifying how the different vaccines affect lymphocyte differentiation, along with widespread naivety that vaccines would terminate the pandemic.

## Discussion

### Back to the Future: Reviewing Prior and Current Approaches to Inform Future Strategies

Now, over 1 year after the first COVID-19 vaccine's emergency approval, we know that the initial intervals between two vaccine doses were too short. Both mRNA vaccines and Ad vectors induce germinal center formation, which is essential for affinity-matured antibody responses, but apparently fail to stimulate long-lived plasma cells (1). mRNA is inherently unstable, leading to transient antigen expression. During B cell differentiation, the consequently brief presence of antigen may preferentially drive differentiation of naïve into memory B cells which, upon reencounter of their cognate antigens from the second dose of mRNA vaccine, mature into short-lived plasma cells. This would explain the rapid waning of antibody responses following mRNA vaccination, and the lack of specific B cell responses upon a boost with a variant-specific mRNA vaccine. Ad vectors, which persist at high levels for weeks (until infected cells are eliminated by CD8+ T cells) would be expected to induce sustained immune responses (6). Nevertheless, available evidence indicates that SARS-CoV-2-specific antibody titers after Ad vector vaccination decline over time too–although not quite as rapidly as those induced by mRNA vaccines. Whether and if Ad vector vaccines evade “original antigenic sin,” allowing variant-specific boosting, remains to be investigated. It also needs to be established whether repeated use of the same Ad vector backbone continues to recall insert-specific antibody responses, or whether vector-neutralizing antibodies will start to impact production of the vaccine antigen and the vector's immunogenicity.

The longevity of protective immunity by other types of COVID-19 vaccines (i.e., the NovaVax Spike protein nanoparticles, or the Chinese killed-virus vaccines) remains uncertain, but the curious finding that the very different mRNA and Ad vector vaccine platforms both fail to achieve the sustained efficacy of other licensed vaccines (e.g., against childhood infections, yellow fever, or smallpox), warrants reflection.

Seasonal human coronaviruses have circulated for centuries, and likewise fail to induce prolonged immunity, but rarely causing severe illness. Notably, although neutralizing antibodies to these viruses fail to cross-react with SARS-CoV-2, the epitopes for other adaptive immune effectors, notably T cells, are shared and an individual's recent infection with seasonal coronaviruses may explain the considerable variability in COVID-19 severity. A better understand of this type of protection may allow design of strategies tailored to confer prolonged protection against severe COVID-19, whether with current or future vaccines.

SARS-CoV-2 will not be eradicated. Variants that are even more transmissible and better equipped to evade immune responses will doubtlessly evolve, triggering new waves of infections. Eventually populations will achieve, via a mixture of infection and vaccination, the same immunological balance with SARS-CoV-2, as with seasonal coronaviruses. The concept that one can vaccinate the world every 4–6 months against COVID-19 is unreasonable. Instead, candid discussions about the future of SARS-CoV-2 vaccination are needed.

The first question that needs to be addressed, is the target population for booster immunizations. Should everyone, including infants, be vaccinated repeatedly or should booster vaccines be reserved for the most vulnerable such as the elderly and individuals with underlying diseases? Children very rarely develop serious or long COVID-19 and might be better off acquiring immunity by natural infections. The value of *repeated* vaccination is also questionable for healthy young and middle-aged adults, especially as natural infections induce stronger and more sustained protection against further infections than mRNA vaccines (7). Vaccines are, however, vital for the elderly and vulnerable, as brutally illustrated by the much higher recent death rate in Hong Kong (with low vaccination rates of care home residents) than South Korea (with its elderly extensively vaccinated). Here the key question, poorly answered by the vaccine trials to date, is: “Which vaccine or combination of vaccines best promotes prolonged immunity against severe disease?”

Secondly, turning to the future, we should mainly contemplate the possibility that the focus, to date, on vaccines that induce neutralizing antibodies directed against the highly variable receptor binding domain of the S1 protein may have been misplaced. Seeking neutralizing antibody responses against the conserved S2 fusion protein may result in more robust protection against variants. Including additional, more conserved viral proteins, may, through CD8+ T cells or non-neutralizing antibodies, increase breadth and potentially duration of protection.

Different vaccine platforms are being tested as vehicles for SARS-CoV-2 antigens, and mucosal routes of immunization are being explored. These efforts may already result in vaccine regimens that are able to induce broader and more durable immune responses.

Nevertheless, for the present, we must accept that although the current shots were highly effective at blunting the pandemic's death toll, they do not offer a long-term solution. More R&D is needed to not only determine how they might be better deployed but also, to develop vaccines able to induce sustained protection against current and future variants.

## Author Contributions

All authors listed have made a substantial, direct, and intellectual contribution to the work and approved it for publication.

## Funding

This work was supported by Virion Therapeutics, LLC.

## Conflict of Interest

HE has the following disclosures: Co-founder of Virion Therapeutics, Advisor roles (board or consultancy) with: Freelance, Inc, Takeda, Biogen (board), Regenxbio, Ring Therapeutics (board), Canine Rabies Treatment Initiative (board). DL has the following disclosures: Advisory roles (board or consultancy): Accelerate, Antabio, Centauri, Entasis, GenPax, GSK, Meiji, Menarini, Mutabilis, Nordic, Paion, ParaPharm, ParaGraf, Pfizer, QPEX, Shionogi, Summit/Discuva, Sumitovant, T.A.Z., VenatoRx, Wockhardt, Zambon; was on the Russian Direct Investment Fund's Advisory Board for the Sputnik V but resigned following the invasion of the Ukraine. Paid lectures: bioMérieux, Beckman Coulter, Cardiome, GSK, Hikma, Merck/MSD, Menarini, Nordic, Pfizer and Shionogi. Relevant shareholdings: Dechra, GSK, Merck, Pfizer and Perkin Elmer amounting to <10% of portfolio value; Share options: T.A.Z. He also has nominated holdings in Avacta, Diaceutics, Destiny, Evgen, Faron, Genedrive, Poolbeg, Renalytics, Saietta, Synairgen and Verici through Enterprise Investment Schemes but has no authority to trade these shares directly. SC has the following disclosures: COO, Virion Therapeutics, Stock, Nektar Therapeutics.

## Publisher's Note

All claims expressed in this article are solely those of the authors and do not necessarily represent those of their affiliated organizations, or those of the publisher, the editors and the reviewers. Any product that may be evaluated in this article, or claim that may be made by its manufacturer, is not guaranteed or endorsed by the publisher.

## References

[1] Giannotta G Giannotta N. mRNA COVID-19 vaccines and long-lived plasma cells: a complicated relationship. Vaccines. (2021) 9:1503. 10.3390/vaccines9121503 34960249 PMC8703557

[2] Bergwerk M Gonen T Lustig Y Amit S Lipsitch M Cohen C. COVID-19 breakthrough infections in vaccinated health care workers. N Engl J Med. (2021) 385:1474–84. 10.1056/NEJMoa2109072 34320281 PMC8362591

[3] Kuhlmann C Mayer CK Claassen M Maponga T Burgers WA Keeton R. Breakthrough infections with SARS-CoV-2 omicron despite mRNA vaccine booster dose. Lancet. (2022) 399:625–6. 10.1016/S0140-6736(22)00090-3 35063123 PMC8765759

[4] Gagne M Moliva JI Foulds KE Andrew SF Flynn BJ Werner AP. mRNA-1273 or mRNA-Omicron boost in vaccinated macaques elicits comparable B cell expansion, neutralizing antibodies and protection against Omicron. bioRxiv. [Preprint]. (2022). 10.1101/2022.02.03.479037 PMC8947944 35447072

[5] Zheutlin A Ott M Sun R Zemlianskaia N Rubel M Hayden J. Durability of protection against COVID-19 breakthrough infections and severe disease by vaccines in the United States *medRxiv*. [Preprint]. (2022). 10.1101/2022.01.05.22268648 PMC9505933 36146536

[6] Tatsis N Fitzgerald JC Reyes-Sandoval A Harris-McCoy KC Hensley SE Zhou D. Adenoviral vectors persist in vivo and maintain activated CD8+ T cells: implications for their use as vaccines. Blood. (2007) 110:1916–23. 10.1182/blood-2007-02-062117 17510320 PMC1976365

[7] Gazit S Shlezinger R Perez G Lotan R Peretz A Ben-Tov A. Comparing SARS-CoV-2 natural immunity to vaccine-induced immunity: reinfections versus breakthrough infections. medRxiv. [Preprint]. (2021). 10.1101/2021.08.24.21262415

